# Conditions of negation formation in children of early age with down syndrome

**DOI:** 10.1192/j.eurpsy.2021.550

**Published:** 2021-08-13

**Authors:** E. Sedova, V. Stepanova, Z. Gardanova

**Affiliations:** 1 Psychological-social Department, Pirogov Russian National Research Medical University, Moscow, Russian Federation; 2 Psychology, Charitable Fund Downside Up, Moscow, Russian Federation

**Keywords:** Down syndrome, parent-child relations, negation, children with special needs

## Abstract

**Introduction:**

According to Vygotsky, children with special needs follow the same trajectory of development as normally developing children, although some of the skills can be observed in a later period. This statement can be implemeted to the children with Down syndrome. The number of such children in Russia is around 25 thousand.

**Objectives:**

The aim is to study the conditions of negation formation in children with Down syndrome.

**Methods:**

The sample consisted of 22 dyads of children with Down syndrome of 24-36 months old and their mothers. The research methods included: parents’ questionnaire; analysis of problematic situations; Tkacheva’s inventory Parent’s Psychological Type; Varga &Stolin Inventory of Parental Attitude; Toronto Alexithymia Scale, Bass-Darky Hostility Questionnaire, Leonhard-Schmieschek Test, Spielberger’s Test Anxiety Inventory.

**Results:**

Firstly, we have studied how a child expresses his or her negative reaction: whether he or she uses a gesture or a sound for “no” or reacts with the whole body. According to those results we have divided the sample into two groups and then have compared them. The research shows the connection between mother’s aggressiveness and formation of the child’s negation reaction (gesture/sound or the whole body) as well as differences in the level of alexithymia and anxiety: all the characteristics are lower in the first group.

**Conclusions:**

Mothers of the children with Down syndrome demonstrate a high and a medium level of anxiety. However, the mothers of the children who expresses negation with a gesture/sound show a lower anxiety level comparing with the mothers whose children react with the whole body.

